# Developing the ecological scientist mindset among underrepresented students in ecology fields

**DOI:** 10.1002/eap.2348

**Published:** 2021-06-28

**Authors:** Gillian Bowser, Carmen R. Cid

**Affiliations:** ^1^ Department of Ecosystem Science and Sustainability Warner College of Natural Resources Colorado State University Fort Collins Colorado 80523 USA; ^2^ School of Arts and Sciences Eastern Connecticut State University 83 Windham Street Willimantic Connecticut 06226 USA

**Keywords:** applied ecology, ecology mentoring, education interventions, environmental workforce, field experience, training diverse ecologists, underrepresented minorities in science

## Abstract

How do students discover ecology? Answering this question is essential for diversifying the environmental workforce because scientific disciplines, such as ecology, are often not discovered until students enter academia and are exposed to different disciplinary options. Ecology, and many of the environmental sciences, have persistent and alarmingly low numbers of underrepresented minorities (URM; African American, Hispanic American, Native American, and Pacific Islanders), while other science and technology fields have shown progress in diversification. Why does such underrepresentation persist in environmental disciplines? Social factors such as sense of belonging, science identity, implicit biases, and stereotypes all have been explored and are known to influence the participation of URM students in science. The unique role of the field experience in environmental sciences as a “rite of passage” and “authentic” research experience is one important influence on how URM students experience ecology. Interventions using social elements such as belonging and sense of place are demonstrated ways to broaden participation particularly in environmental science fields, yet dramatic underrepresentation still persists. Here we review known factors affecting and enhancing the recruitment and retention of URMs in the sciences and focus on comprehensive strategies shown to be effective recruiting URM students into the environmental workforce.

## 1 Introduction

The diversity within the environmental workforce does not reflect the human communities they serve. To date, minorities and persons of disabilities (URM) remain significantly underrepresented proportional to their numbers in the United States population in Science Technology Engineering and Mathematics (STEM) fields (NCSES 2021). Progress on increasing participation for minorities continues to lag in the earth sciences, including geosciences, ecology, and other natural resource fields, while the representation of white women in those same sciences has increased consistently (Ortega et al. 2006, Taylor 2017, NCSES 2021).

How do URM students discover ecology disciplines? As background, in 1992, the Ecological Society of America (ESA) surveyed the diversity of its membership (Lawrence et al. 1993*a*). At the time, the data showed <5% ethnic diversity in ESA members. Approaching the ESA centennial in 2015, more than 20 yr later, ESA membership had reached at most 9% (Beck et al. 2014). The same survey also assessed how members had first become interested in ecology (Lawrence et al. 1993*b*). Sixty percent of the respondents had discovered ecology at an early age by participating in some guided field experience program and 32% were introduced to the discipline by a college professor. More recent data in 2015 continued to show the importance of mentors and field experiences in their discovery of ecology.^4^

Minority communities are not less interested or engaged in the environmental issues associated with ecology as a whole (Leiserowitz et al. 2018). The impacts of global climate change and associated environmental problems tend to be concentrated in communities of color (Otto et al. 2017) and the juxtaposition of poor communities of color and environmental toxins laid the foundations for environmental justice research, policies, and activism (Bullard 2018, Pearson and Schuldt 2018). Recent data indicate that environmental awareness is steadily increasing among ethnic minorities (Leiserowitz and Akerlof 2010, Taylor 2017), and that the underrepresentation of minorities in ecology is not from some inherent disinterest in the environment for those minority populations.

The need to make the environmental workforce match the increasingly diverse demographics of human communities has prompted research inquiry on what factors specifically affect URM students' career choices in environmental science fields. For environmental careers, elements influencing URM engagement include many different factors such as family support (Armstrong et al. 2007), participation in guided experiences in nature appreciation (whether in urban or more natural field sites; Aloisio et al. 2018), exposure to careers in ecology (Morales et al. 2020), connecting environmental study in some way to interests in solving local and global community problems that affect minorities (Bowser et al. 2020), and field research experiences as high school or college students in any type of environmental setting (Flowers et al. 2016, Burrow 2018, O'Connell et al. 2018, Beltran et al. 2020). All of these experiences can lead URM students into further study of the environment. However, even with engagement by good mentors, URM students face additional barriers that include a lack of connection between the scientific field research opportunities, and life in their own communities (Hugo et al. 2013) in ways that do not promote comfort and engagement in studying environmental issues of interest (Miriti 2019).

Here we focus on how to infuse cultural and social elements as a part of ecological education objectives to engage URM students in ecology and environmental science. Our objectives are to (1) review foundational literature and research focused on URM participation in ecology, (2) compare single factor (focus on science) with multifactor (integrated with social factors) ecological programming approaches, (3) provide a framework and guidelines for adopting comprehensive and innovative approaches to URM engagement in applied ecology fields, and (4) suggest future strategies for research and practice.

**Fig. 1:**
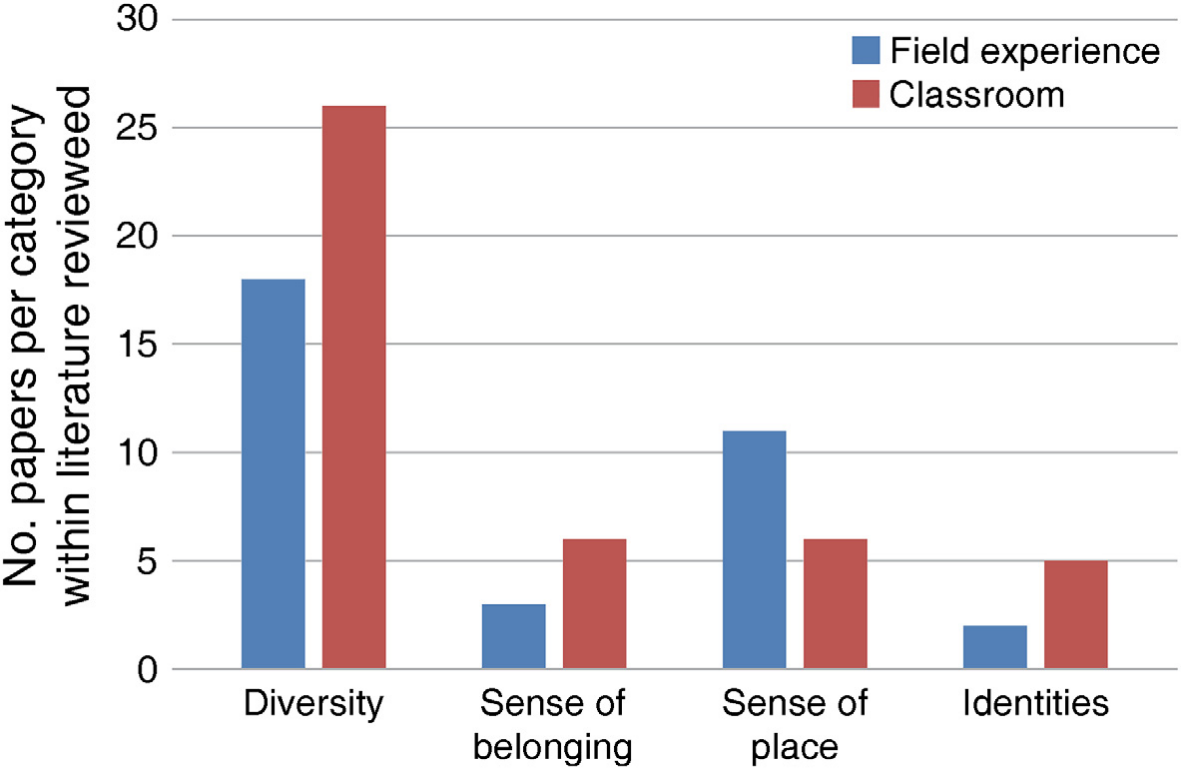
Literature review results for field experience (*n* = 31) and classroom/theory papers (*n* = 43) focusing on (1) diversity, (2) sense of belonging, (3) sense of place, or (4) identities in relation to STEM education are shown on the *x‐*axis and number of papers per category within the literature reviewed are shown on the *y*‐axis).

Our literature review focused on elements identified by ecologists as important to their own professional development such as (1) the “rite of passage” of the field experience, (2) sense of belonging to a group and the outdoor culture, (3) sense of place in ecology instruction, and (4) identifying as a scientist or field ecologist (Fig. 1, Appendix S1). Research on field experience programs has shown that active learning can help broaden participation in environmental education, but the focus has been primarily on increasing student self‐efficacy and less on integrating the human dimensions into developing a scientist identity (Ballen et al. 2017, O'Connell et al. 2018). A wealth of research on identity, belonging, and place attachment exists in the social science literature (Yeager and Dweck 2012, for example); however, these approaches have not been applied to the field experience and most of the relevant papers lie on the boundaries of ecology, science learning, and URM recruitment.

We have structured our discussion in three parts: *Part 1: The Field Experience*; *Part 2: Brief Interventions*; and *Part 3: Identity and Mindsets*. *Part 1* explores the role of the field experience as an introduction to the discipline of ecology and the current widespread underlying emphasis on elements of learning ecological science (as reviewed in Smith et al. 2019). *Pa* *rt 2* outlines single and multiple factor interventions that introduce URM students to science in different ways and how such approaches lead to a combination of science learning targets and social science metrics such as belonging and identity. Finally, *Part 3* discusses the combination of social elements with core ecological concepts that can be structured in field experiences, can lead to ecological scientist mindsets that connect across multiple cultures around environmental learning and a sense of identity as a scientist.

### 1.1 Part 1: The field experience

Many academic and professional ecologists recall their first field experience as the moment when they felt that a career in field‐based ecology was for them (Bowser et al. 2012). Such field experiences are often part of the broader impacts of ecological research to foster the social connections that play important roles in advancing ecological careers. Over the last three decades, the National Science Foundation's Broader Impacts requirements for research proposals (NSF 2020), Ecological Society of America's (ESA) environmental education professionals, and the American Association for the Advancement of Science's Vision and Change initiative, have all emphasized addressing the human dimensions in ecology (Brewer and Smith 2011, Cid and Pouyat 2013, Cid and Bowser 2015, Skrip 2015, Austin and Smith 2018, Berkowitz et al. 2018, Hansen et al. 2018). The National Science Foundation defines Broader Impacts as “…the potential to benefit society and contribute to the achievement of specific, desired societal outcomes…” (NSF 2020). Criteria for broader impacts as measures of society impact are often used to express some connection to diverse audiences or without any sense of belonging to the complex multicultural nature of society (Skrip 2015). However, in most field experience programs the integration of broader impacts and associated social interactions to create and promote trust and self‐confidence in students is often lacking or needs development.

Sense of belonging is often overlooked as a part of the broader impact spectrum of a mindset of resilience where URM students develop an “…emotional response to academic or social challenges that is positive and beneficial for development…” (Yeager and Dweck 2012). The success of using “sense of belonging” interventions to improve recruitment and retention of URM students in science fields has also documented development of leadership skills (Walton and Cohen 2011). Similar interventions can be applied to create bridges for URM students to pursue a broad array of ecological science careers (Kudryavtsev et al. 2012, Russ et al. 2015, Mourad et al. 2018, Halliwell et al. 2020).

The latest literature suggests concern that the persistent underrepresentation of minorities in ecology is due to the culture of ecology as a discipline (Rainey et al. 2018, Miriti 2019). Since environmental study often requires field work, research has focused on internal factors associated with the rites of passage connected to the field experiences that create a sense of identity and belonging as an ecologist (Morales et al. 2020). Field experiences, whether a formal field class or research experience in outdoor settings, are considered a critical part of how students choose to enter the environmental workforce (Kloser et al. 2013, Flowers et al. 2016, Thompson et al. 2016, Fleischner et al. 2017, Berkowitz et al. 2018, Beltran et al. 2020). Many programs require students to have research experiences and professional organizations often rate these experiences as critical for employment (Haynes and Jacobson 2015). The field experience for applied ecology professions is a pivotal experience that can be a barrier, or an enabler, of student participation especially for underrepresented groups. The connection between appreciation for issues of global environmental concern (climate change, environmental justice for example) and the pursuit of ecological study at the college level has not been effectively made for most URM students (Taylor 2017, Hansen et al. 2018).

The most comprehensive effort to date to elevate the human dimensions in field experience programs as well as in undergraduate ecology curricula has been the 2018 ESA endorsement of the Four‐Dimensional Ecology Education curricular framework (4DEE) (Klemow et al. 2019). The 4DEE framework promotes the discussion of human–environment interactions (human impact and codependence) in the teaching of all ecological topics in undergraduate and graduate environmental coursework (Ecological Society of America 2020). The 4DEE stresses the importance of science communication, field work experience and data/technology skills as consistent and critical parts of such instruction. The 4DEE curriculum focuses on what content to cover in college courses to better address the needs of societal‐environmental problem‐solving (Smith et al. 2019). Bringing human dimensions into ecological studies and field experiences is complex and, outside of urban field experiences, does not appear to be a widely used in ecology and potentially could engage more URM students (Russ et al. 2015, Taylor 2017, Mourad et al. 2018).

### 1.2 Part 2. Brief interventions

**Fig. 2:**
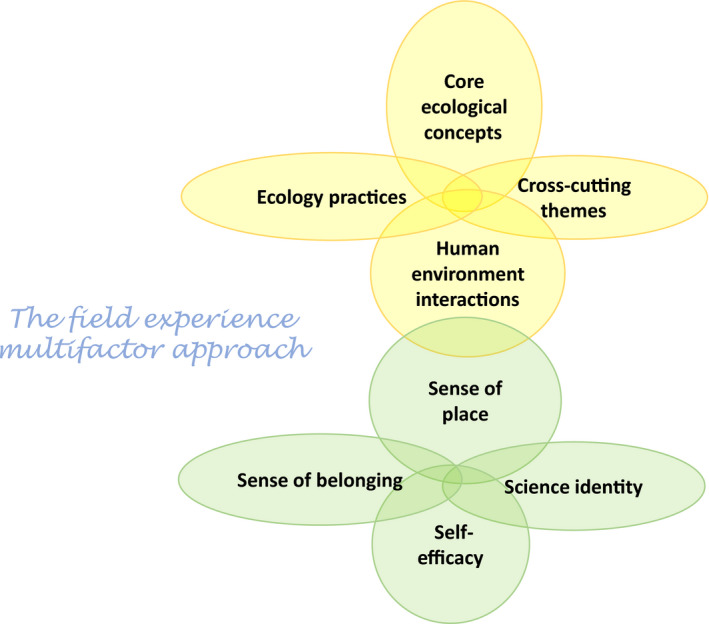
Multiple factor interventions. Ecological science elements (yellow; as outlined in current ESA‐endorsed 4DEE curriculum) integrate with social science elements (green) as part of the ecology field experience to provide intercultural connections.

The literature indicates that brief interventions (short interval) can have an impact on science learning gains for students and promote the recruitment and retention of underrepresented groups in the sciences (Walton and Cohen 2007, 2011). Such brief interventions, as defined by Walton and Cohen (2011) focus on a single factor intervention (such as a field experience) or combine multiple interventions that include both class‐based science learning and semester‐long project‐based teamwork (Fig. 2). Single factor interventions, however brief (i.e., not sustained over long periods), can have significant impact on student performance in science; multifactor interventions affect long term retention and identity as a scientist (Walton and Cohen 2011, Davis et al. 2012, Halliwell et al. 2020).

Multifactor interventions that blend sense of place, sense of belonging, team building, and other social elements with science are difficult to execute in the field. Emphasizing a balance of science and project‐based learning approaches (Thompson et al. 2016, Burrows 2018, Mourad et al. 2018, Halliwell et al. 2020), where the team itself is an integral part of the process, can be fundamentally different than a research experience for undergraduates (REU) experience. Ecological field experiences with an emphasis on multifactor interventions can change how all students learn (Singer 2019) and improve URM student participation (Kudryavtsev et al. 2012, Russ et al. 2015, Carpi et al. 2017).

**Fig. 3:**
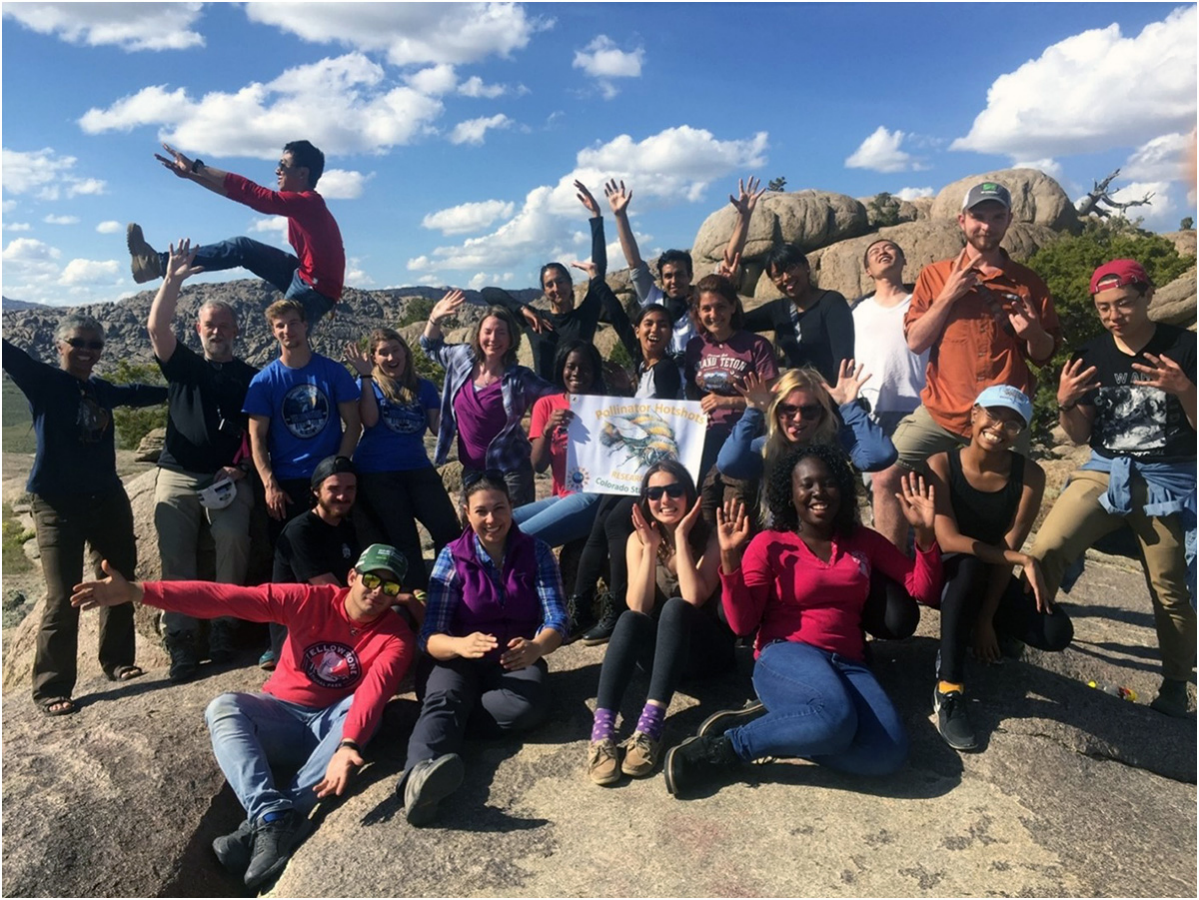
Developing sense of place in project teams conducting research in the Rocky Mountain Science Students Network (RMSSN) academy program through highlighting cultural connections that enhance student and sense of belonging in science. Photo credit: Gillian Bowser.

Multifactor interventions can be relatively short and intense in a field or professional setting and result in high URM recruitment that often is not the case for longer interventions and research experiences (Diaz Eaton et al. 2016). An example of a multifactor intervention is the Rocky Mountain Science and Sustainability Network's (RMSSN) academy, started with a five‐year grant from the National Science Foundation to G. Bowser and M. A. Brown in 2009 (NSF DBI#0956059; DBI#1624191), to form a research coordination network in undergraduate biology education (RCN‐UBE). RMSSN was an informal learning program to teach ecology and environmental sciences, with several distinct goals: (1) engage underrepresented students in a field experience to have them build capacity in designing experiments around core ecological concepts, (2) provide a team‐based experiences to build confidence in understanding data design and scientific inquiry; (3) build a sense of belonging for all students to be a part of science inquiry, and (4) provide critical thinking skills and data analysis around a sense of place associated with applied ecological principles. Connecting local ecological issues to global issues, and engagement in citizen science has been a valuable component and a critical assessment component for students post RMSSN academy (Gretzel et al. 2014, Halliwell and Bowser 2019, Halliwell et al. 2020; Fig. 3).

The RMSSN multifactor intervention starts with the application process that prioritizes students with a leadership background and interest in global environmental issues over grade point average or other metrics. Applicants share how they view environmental issues as well as their own assessment of what a leader is. The RMSSN application avoids questions that put emphasis on field skills (e.g., have you ever been camping?) or easy‐to‐learn skills (do you know how to set up a tent?). Such reframing of questions about skills can raise the applicants' perception of their ability to be leaders in a novel or unfamiliar setting. Using social skills, such as the ability to work with or lead a group of peers (e.g., have you ever led a team of your peers on a novel adventure?), as the core metric for a field experience, elevates a different pool of applicants who see themselves as leaders regardless of their experience in actual field settings. This first step is critical, yet often is overlooked, as the application process itself can discourage students from even considering the experience and/or the field of ecology itself. These early interventions can bring different students into ecology by focusing on skillsets they can define themselves (I see myself as a leader because…) regardless of whether the student is from an urban apartment building or rural farming community.

Another multifactor intervention program, now 25 years old, is the Ecological Society of America's Strategies for Ecology Education, Diversity and Sustainability (SEEDS) program. The award‐winning SEEDS program recruits primarily upper‐level science majors with leadership potential from long established university‐based SEEDS student chapters and clubs. SEEDS students participate in group field trips to an important ecological site with faculty mentors, attend a leadership gathering at the annual ESA conference, where they are assigned mentors, and engage in scientific and social activities. A series of short multifactor interventions (3–4 d to 1 week) take place over a year that include developing professional skills, group projects, social components, reflection, and sharing (Mourad et al. 2018). To date, one measure of the success of the SEEDS program is that 71% of the alumni have persisted in the environmental sciences and pursued environmental careers (Ahern‐Dodson et al. 2020).

Moving beyond single‐factor focus (on science) and incorporating social and cultural elements (belonging, identity) combined with science learning is well documented to shift student academic success in the sciences and increase their retention in science as a whole (Miriti 2020). Important steps toward diversifying the ecological disciplines thus include multifactor interventions that focus on URM students' sense of identity and belonging through cultural connections as well as field experiences.

### 1.3 Part 3: Identity and mindsets

In this paper, our goal has been to understand how to maximize the number of URM students who develop an ecological scientist mindset, i.e., a mindset that enhances their ability to understand ecological principles, engage in ecological research, and pursue professional careers in ecology. Ecology focuses on observing patterns in nature, species interactions, and environmental change (Reiners et al. 2013, Tewksbury et al. 2014, Barrows et al. 2016, Reiners 2016, McKeon et al. 2020). An ecological mindset is an outcome when observations of patterns are combined with developing a sense of identity and belonging within the field of ecology. As students build confidence in their ability to conduct ecological science, they begin to identify as scientists and expand their mindset toward addressing global environmental issues (Davis et al. 2012).

There are at least five elements to the effective multifactor intervention to recruit and retain URM students in ecology and develop their ecological scientist mindset: (1) recruiting students who have leadership potential and are interested in making a difference in society, (2) spending time developing the team spirit and sense of community with structured project‐based learning including social exercises, (3) picking project ideas that can easily be connected to the cultural values and interests of URM students (Miriti 2019), (4) connecting the experience to different senses of place and of belonging for diverse cultures, and (5) incorporating innovative technology (Palumbo et al. 2012) or art visualization (Ellison et al. 2018) valued by the student age groups and cultures.

Field ecological research requires that ecologists be able to quickly integrate data collection and analysis methods to cope with unexpected environmental circumstances, disturbances and a wide range of unanticipated challenges. Thus, the training of all ecologists needs to promote the development of a “resilience mindset.” Yeager and Dweck (2012) define a resilience mindset by four characteristics: (1) student goals, what drives students engagement in project‐based learning using the students' own goals toward learning (eagerness to learn); (2) beliefs about effort, do they perceive themselves to have the natural talent needed, or perceive they lack some skill and thus fail to even engage in the experience; (3) attributions, ability to handle setbacks as part of the experience; and (4) learning strategies, unknowns and personal effort (try harder or give up).

Resilience mindsets respect the incoming student's culture and are critical dimensions of belonging and the lived experience, whether in urban or rural settings (Fleischner et al. 2017, Aloisio et al. 2018). When students self‐organize a project, they can incorporate such lived experiences into research designs or team assembly. Yet, while such projects may have great risk of failure or non‐significant results, the self‐organized, project‐based, learning framework itself can teach resilience and provide opportunities as well as develop observation skills that help students identify as being a scientist. Resilience is an important component of an ecological scientist mindset, especially when rooted in a sense of belonging and connections beyond just the science outcomes of a project; “…we learned how to observe the data and despite our inability to locate [the organisms]…we feel a special connection to the park itself.” (student observer remarking on why Yellowstone was now a special place for her [Halliwell and Bowser 2019]).

Belonging, resilience, and sense of place can be enhanced using short creative interventions. Developing these traits can provide the bridge needed for URM students to connect with ecology, and create a sense of purpose that identifies with internal and cultural desires to make a difference. Such bridges are also important at pre‐college levels and can help URM students enter college with an interest in the ecological sciences, already equipped with a sense of identity and belonging as an ecologist (Torres and Bingham 2008). Sense of place and sense of identity can be seen as elements of an ecological identity “Ecological identity focuses one's attention on environmental activities, green infrastructure, ecosystems, and biodiversity, including in urban places” (Kudryavtsev 2016). Identity, resilience, and ecological mindsets all lead to gains in student learning; retention in the discipline and movement into professional careers.

**Fig. 4:**
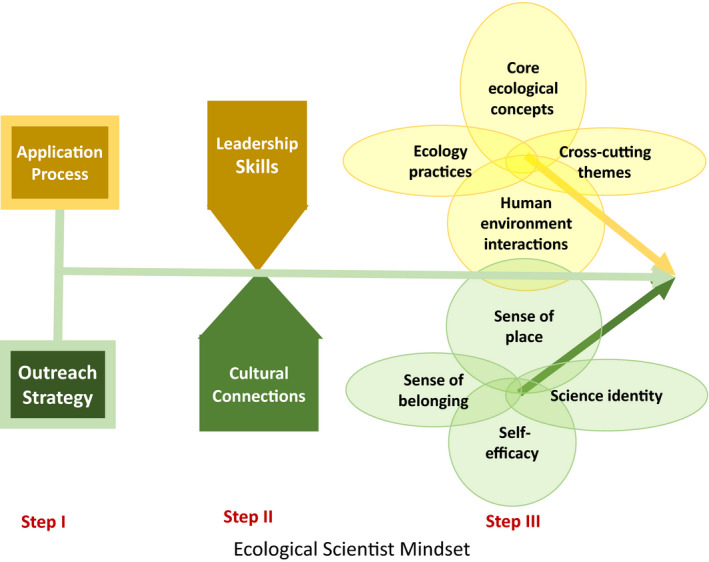
Guide to multifactor brief interventions that link culture and science to create an ecological scientist mindset. The three steps of planning a field experience include: Step I, recruitment process; Step II, preparation of selected students; and Step III, field experience in which ecological concepts are combined with human dimension concepts. These three steps connect, as indicated by arrows, and culminate in an ecological scientist mindset.

How do you hook students into ecology for life? To create an ecological scientist mindset for an audience of URM students requires a three‐step intervention (Fig. 4) that starts with the recruitment process (applications are focused on finding students having leadership skills without a need for prior field experience that would eliminate many URM students [Step I]), continues with preparation of selected students to increase and strengthen their individual and cultural sense of place and belonging (Step II), and culminates in an inclusive field experience (Step III) that raises confidence and self‐efficacy, leading to an ecological scientist mindset (Table 1). Interventions that focus on retention of URMs in ecology can then be assessed for effectiveness across different cultural and racial ethnicities that together weave a pathway to an ecological mindset, centered in the student's cultural and/or racial identity.

**Table 1:** Student research program logic model for brief 4Cs designed interventions to bridge culture and science and enhance broader impacts to in student field research experiences.

Intervention step	Desired outcome	Measures of success
Step I: Recruitment	Comfort: URM students apply to program.	Increase in number of applications from URM students.
Step II: Student preparation	Connection: through assigned online research, all students develop cultural connection to proposed field site and engage their leadership skills in preparing for field experience.	Increased sense of place in student participants, especially for URM students. Students can articulate their cultural connection to the proposed research project.
Step III: Field experience	Confidence: students gain ecological knowledge and skills in performing field research to enhance their training as ecologists.	Increased self‐efficacy through ability to design and test hypotheses in the field site. Increased sense of belonging to the community of ecologists, especially in URM students.
Ultimate goal: Ecological scientist mindset assessment	Capacity: Natural history observational skills, skills in designing and testing ecological hypotheses are developed.	Students, especially URM students, increase their ability to perform ecological work and continue to pursue environmental careers.

Notes

The 4Cs are comfort, connection, confidence, and capacity. All students accepted in the program need to be assessed pre‐field experience for measures of success indicated.

The RMSSN intervention focused on creating resilience mindsets that involves URM students in the use of easily accessible and doable data collection techniques, utilizing cell phone technology, cultural stories, art, and citizen science. In contrast, the SEEDS intervention focuses on creating ecological identity through professional development, peer activities, and leadership in the sciences. The connecting principle for both interventions is social: the students need a sense of purpose and the ability and confidence to make a difference through their own actions as future scientists. Introducing social elements and sense of belonging attracted diverse students and created an environment within which they could succeed.

An exclusive focus on recruitment is not the solution for the underrepresentation in the sciences (Brewer and Smith 2011, Austin and Smith 2018). Diversity efforts need to be more than the individual faculty or administrators' passion. Successful interventions should be rooted in robust scholarship and evidence, not just notions about the barriers URM students might face in science. We see an emerging framework that focuses on enhancing four social characteristics (4Cs) in the targeted student population: comfort with ecological field experiences; connection to the study site through sense of place preparation; confidence through team building exercises and fieldwork engagement; and capability through comprehensive field research programs (Table 1). Building these interventions within existing field experience programs can help create an inclusive “rite of passage” experience for URM students and generate and maintain interest in the field of ecology early in their academic careers and into their professions.

## 2 Framing Solutions

Field projects and experiences that strive to reach diverse audiences (often school children) or engage historically black colleges and universities (HBCU) often fail or underperform because “one‐way provisioning of science information [that]… Mostly will not work…” (Skrip 2015). Barriers to underrepresentation by a specific minority group can be subtle and hard to detect. The integration of cultural competency with traditional pedagogy needs to occur in ecology, especially in field settings, such as research stations or field camps, where the sense of belonging and identity experienced by majority demographic can create barriers for those from diverse cultural groups. Field experiences work when they are combined with team‐building exercises that create a sense of belonging and provide that rite of passage for *all* students leading toward a lifelong passion for ecology (Thompson et al. 2016, Halliwell et al. 2020). The sense of place component works when combined with a connection to the students' family values/cultural heritage and upbringing that also creates a sense of belonging for participating students: “My people were here too and I am not the first!” (African American student response on discovering the story of Buffalo Soldiers in Yellowstone National Park [Halliwell and Bowser 2019]). Recent acknowledgements from land grant institutions of the prior sovereign nations lands upon which they sit represent one path that highlights the value of telling the stories that respect the importance of place (Lee and Ahtone 2020). Such stories help reconnect all students with the culture of the landscapes providing them with perspectives of the many diverse cultures embedded with the same landscape. Similar acknowledgments in field locations create that same connection to the cultural landscape, woven tightly around the ecological processes themselves and creating special bonds for different cultural and demographic groups. Groups that may have called the landscape home or trace historical routes or passages find meanings that connect to that sense of belonging and place. Mentoring models for diversity advocates and allies need to include stories that combine a sense of belonging and a sense of place that acknowledges the diversity of students entering academia today.

How can we ensure URM students will discover ecology and stay engaged? One way to make progress is to focus first on the student and then on the science. Developing resilience in students during that first field experience allows URM students to explore and discover new things, but also provides the tools they need to succeed as a minority in a majority cultural setting, even without explicit mentor or peer support (Ballen et al. 2017, Carpi et al. 2017, Hansen et al. 2018, Beltran et al. 2020).

Many URM faculty or professionals who received their academic degrees at predominately white institutions can clearly remember the first URM professor they had in a class regardless of the science discipline (Bowser et al. 2012). Similarly, they can also remember the first field experience where they felt welcomed as both a minority and ecological professional and thus, officially part of the “club” (Cid and Bowser 2015, Cid and Brunson 2020). Resilience mindset allows URM students to deal with, not just the process of discovery and exploration in sciences, but also the sense of belonging and participating in a rite of passage for ecological fields even if there is no one who looks like them in that field experience setting.

**Fig. 5:**
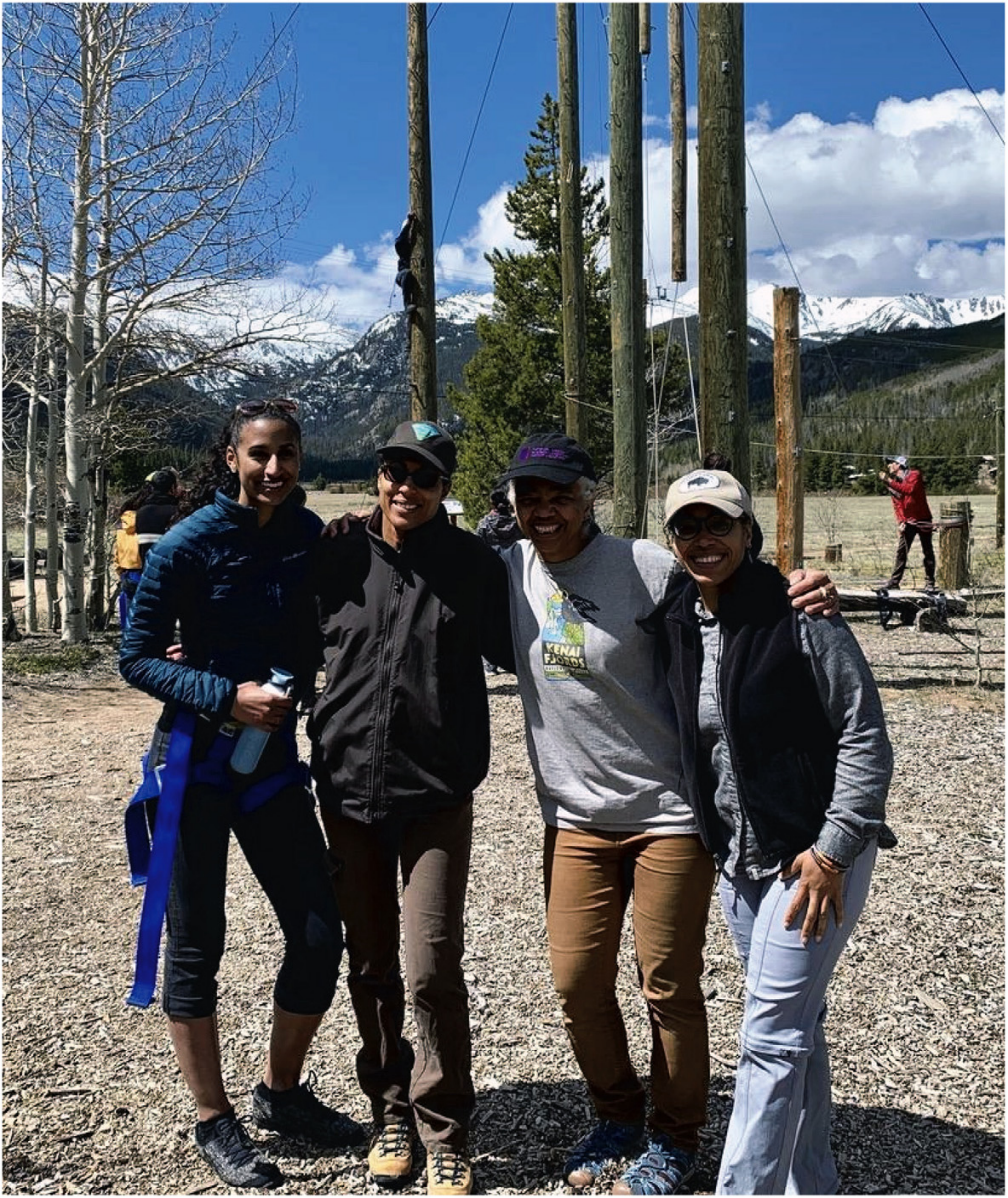
Fostering the ecological scientist mindset broadens the “club” to include women of color who identify as scientists (RMSSN faculty mentors ‐ left to right: Tashiana Osborne, atmospheric scientist; Nikki Hoffman‐Grant, plant ecologist; Gillian Bowser, wildlife ecologist; Omayra Ortega, mathematician). Photo credit: Gillian Bowser.

Focusing on the social elements (the 4Cs) and leadership mindsets can provide an effective framework for bringing new audiences into ecology despite it being a “discovered” major in academia. Why is this urgent and relevant today? Rapid global environmental change and increasing impacts of wildlife–human disease transfer provide an immediate sense of urgency since the people who are most impacted by these changes are the same who are left out or pushed out of science careers. Such affected groups need to have access to robust scientific data that are provided within their cultural context and by scientists who reflect those same audiences (Fig. 5). Communicating science in a culturally competent and relevant manner has never been more critical, especially as the world seeks solutions for global challenges like the COVID‐19 pandemic and climate change. Developing an “ecological scientist mindset” in all students, regardless of cultural identity, promotes global wellbeing and sustainability. Moving forward, having a science workforce that is not only integrated but works together across cultural spaces and identifies as a science community with shared data knowledge should be our common vision for our students and future practitioners of science.

## Supporting information

Appendix S1

## Footnotes

^4^

https://esa.org/history/biographies/ecological‐richness‐diverse‐contributors‐of‐diverse‐contributions/

## References

[eap2348-bib-0001] Ahern‐Dodson, C. R., T. M. Clark, and J. A. Reynolds. 2020. Beyond the numbers: understanding how a diversity mentoring program welcomes students into a scientific community. Ecosphere 11:e03025. 10.1002/ecs2.3025

[eap2348-bib-0002] Aloisio, J. M., B. Johnson, J. D. Lewis, J. A. Clark, J. Munshi‐South, S. Roberts, D. Wasserman, J. Heimlich, and K. Tingley. 2018. Pre‐college urban ecology research mentoring: promoting broader participation in the field of ecology for an urban future. Journal of Urban Ecology 4:1–9.

[eap2348-bib-0003] Armstrong, M. J., A. R. Berkowitz, L. A. Dyer, and J. Taylor. 2007. Understanding why underrepresented students pursue ecology careers: a preliminary case study. Frontiers in Ecology and the Environment 5:415–420.

[eap2348-bib-0004] Austin, A., and D. Smith. 2018. Vision and change: unpacking a movement and sharing lessons learned. American Association for the Advancement of Science, Washington, DC, USA. https://www.visionandchange.org

[eap2348-bib-0005] Ballen, C. J., C. Wieman, S. Salehi, J. B. Searle, and K. R. Zamudio. 2017. Enhancing diversity in undergraduate science: self‐efficacy drives performance gains with active learning. CBE Life Science Education 16:ar56. 10.1187/cbe.16-12-0344 PMC5749958 29054921

[eap2348-bib-0006] Barrows, C. W., M. L. Murphy‐Mariscal, and R. R. Hernandez. 2016. At a crossroads: the nature of natural history in the twenty‐first century. BioScience 66:592–599.

[eap2348-bib-0007] Beck, C. K., C. S. Boersma, C. S. Tysor, and G. Middendorf. 2014. Diversity at 100: women and underrepresented minorities in the ESA. Frontiers in Ecology and the Environment 12:434–436.

[eap2348-bib-0008] Beltran, R. S., E. Marnocha, A. Race, D. A. Croll, G. H. Dayton, and E. S. Zavaleta. 2020. Field courses narrow demographic achievement gaps in ecology and evolutionary biology. Ecology and Evolution 10:1–13. 10.1002/ece3.6300 PMC7319162 32607142

[eap2348-bib-0009] Berkowitz, A. R., C. Cid, J. Doherty, D. Ebert‐May, K. Klemow, G. Middendorf, T. Mourad, and B. Pohlad. 2018. The 4‐Dimensional Ecology Education (4DEE) Framework. Report to the Ecological Society of America. http:/esa.org/4dee

[eap2348-bib-0010] Bowser, G., S. A. Green, S. S. Ho, and P. H. Templer. 2020. Educating students in solutions‐oriented science. Frontiers in Ecology and the Environment 18:171.

[eap2348-bib-0011] Bowser, G., N. S. Roberts, D. R. Simmons, and M. K. Perales. 2012. The color of climate: ecology, environment, climate change and women of color‐exploring environmental leadership from the perspective of women of color in science. Pages 60–67 *in* D. R. Gallagher, editor. Environmental leadership in practice: a reference handbook. Volume 1. Sage Publications, Thousand Oaks, CA.

[eap2348-bib-0012] Brewer, C., and D. Smith. 2011. Vision and change in undergraduate education: a call to action. American Association for the Advancement of Science, Washington, DC, USA. https://www.visionandchange.org

[eap2348-bib-0013] Bullard, R. D. 2018. Dumping in Dixie: race, class, and environmental quality. Third edition. Routledge, New York, New York, USA.

[eap2348-bib-0014] Burrow, A. 2018. Teaching introductory ecology with problem‐based learning. Bulletin of the Ecological Society of America 99:137–150.

[eap2348-bib-0015] Carpi, A., D. M. Ronan, H. M. Falconer, and N. H. Lents. 2017. Cultivating minority scientists: undergraduate research increases self‐efficacy and career ambitions for underrepresented students in STEM. Journal of Research and Science Teaching 54:169–194.

[eap2348-bib-0016] Cid, C. R., and G. Bowser. 2015. Breaking down barriers to diversity in ecology. Frontiers in Ecology and the Environment 13: 179.

[eap2348-bib-0017] Cid, C. R., and M. W. Brunson. 2020. Engaging faculty in preparing students for non‐academic environmental careers. Frontiers in Ecology and the Environment 18:52–53.

[eap2348-bib-0018] Cid, C. R., and R. V. Pouyat. 2013. Making ecology relevant to decision making: the human‐centered, place‐based approach. Frontiers in Ecology and the Environment 11:447–448.

[eap2348-bib-0019] Davis, E., G. Bowser, and M. A. Brown. 2012. The Global Mindset: engaging multicultural students in multidimensional learning. Pages 891–899 *in* D. R. Gallagher, editor. Environmental leadership in practice: a reference handbook. Volume 2. Sage Publications, Thousand Oaks, CA. 1988–2011

[eap2348-bib-0020] Diaz Eaton, C., D. Allen, L. A. Anderson, G. Bowser, M. A. Pauley, K. S. Williams, and G. E. Uno. 2016. Summit of the research coordination networks for undergraduate biology education. CBE—Life Sciences Education 15:mr1. 10.1187/cbe.16-03-0147

[eap2348-bib-0021] Ecological Society of America 2020. The four dimensional ecology education framework. Ecological Society of America, Washington, DC, USA. https://www.esa.org/4dee/framework

[eap2348-bib-0022] Ellison, A. M., et al. 2018. Art/Science collaborations: new explorations of ecological systems, values and their feedbacks. Bulletin of the Ecological Society of America 99:180–191.

[eap2348-bib-0023] Fleischner, T. L., et al. 2017. Teaching biology in the field: importance, challenges and solutions. BioScience 67:558–567.

[eap2348-bib-0024] Flowers, S. K., K. M. Beyer, M. Perez, and D. B. Jeffe. 2016. Early environmental field research career exploration: an analysis of impacts on pre‐college youth apprentices. CBE‐Life Sciences Education 15:ar67. 27909017 10.1187/cbe.15-11-0230 PMC5132364

[eap2348-bib-0025] Gretzel, U., E. B. Davis, G. Bowser, J. Jiang, and M. A. Brown. 2014. Creating Global Leaders with sustainability mindsets—reflections from the RMSSN Summer Academy. Journal of Teaching in Travel & Tourism 14:164–183.

[eap2348-bib-0026] Halliwell, P., and G. Bowser. 2019. A diverse sense of place: citizen science as a tool to connect underrepresented students to science and the national parks. Mountain Views Cirmount 13:45.

[eap2348-bib-0027] Halliwell, P., S. Whipple, and G. Bowser. 2020. 21st Century climate education: developing diverse, confident, and competent leaders in environmental sustainability. Bulletin of the Ecological Society of America 101:e01742. 10.1002/bes2.1664

[eap2348-bib-0028] Hansen, W. D., et al. 2018. How do we ensure the future of our discipline is vibrant? Student reflections on careers and culture of ecology. Ecosphere 9:e02099.

[eap2348-bib-0029] Haynes, N. A., and S. Jacobson. 2015. Barriers and perceptions of natural resource careers by minority students. The Journal of Environmental Education 46:166–182.

[eap2348-bib-0030] Hugo, R., W. F. Smythe, S. McAllister, B. Young, B. Marring, and A. Baptista. 2013. Lessons learned from a K‐12 geoscience education program in an Alaska native community. Journal of Sustainability Education 05:5.

[eap2348-bib-0031] Klemow, K., A. Berkowitz, C. R. Cid, and G. Middendorf. 2019. Improving ecological education through a four‐dimensional framework. Frontiers in Ecology and the Environment 17:71.

[eap2348-bib-0032] Kloser, M. J., S. E. Brownell, R. J. Shavelson, and T. Fukami. 2013. Effects of a research‐based ecology lab course: a study of non‐volunteer achievement, self‐confidence and perception of lab course purpose. Journal of College Science Teaching 42:72–81.

[eap2348-bib-0033] Kudryavtsev, A. 2016. Developing an ecological identity. https://naaee.org/eepro/blog/sense‐place

[eap2348-bib-0034] Kudryavtsev, A., M. E. Krasny, and R. C. Stedman. 2012. The impact of environmental education on sense of place among urban youth. Ecosphere 3:29.

[eap2348-bib-0035] Lawrence, D. M., M. M. Holland, and D. J. Morrin. 1993a. Profiles of ecologists: results of a survey of the membership of the Ecological Society of America. Part I. A snapshot of survey respondents. Bulletin of the Ecological Society of America 74:21–35.

[eap2348-bib-0036] Lawrence, D. M., M. M. Holland, and D. J. Morrin. 1993b. Profiles of ecologists: results of a survey of the membership of the Ecological Society of America. Part II. Education and employment patterns. Bulletin of the Ecological Society of America 74:153–169.

[eap2348-bib-0037] Lee, R., and T. Ahtone. 2020. Land Grab Universities. Expropriated indigenous land is the foundation of the land grant university system. High Country News, Paonia, Colorado, USA. https://www.hcn.org/issues/52.4/indigenous‐affairs‐education‐land‐grab‐universities

[eap2348-bib-0038] Leiserowitz, A., and K. Akerlof. 2010. Race, ethnicity and public responses to climate change. Yale University and George Mason University, New Haven, Connecticut, USA. Yale Project on Climate Change. http://environment.yale.edu/uploads/RaceEthnicity2010.pdf

[eap2348-bib-0039] Leiserowitz, A., S. Rosenthal, and M. Cutler. 2018. Latinos and global warming's Six Americas. Yale Program on Climate Change Communication. Yale University, New Haven, Connecticut, USA.

[eap2348-bib-0040] McKeon, S., L. Weber, A. J. Adams, and T. L. Fleischner. 2020. Human dimensions: natural history as the innate foundation of ecology. Bulletin of the Ecological Society of America 101:e01656.

[eap2348-bib-0041] Miriti, M. 2019. Nature in the eye of the beholder: a case study for cultural humility as a strategy to broaden participation in STEM. Education Science 9:291.

[eap2348-bib-0042] Miriti, M. 2020. The elephant in the room: race and STEM diversity. BioScience 70:237–242.

[eap2348-bib-0043] Morales, N., K. B. O'Connell, S. McNulty, A. Berkowitz, G. Bowser, M. Giamellaro, and M. Miriti. 2020. Promoting inclusion in ecological field experiences: examining and overcoming barriers to a professional rite of passage. Bulletin of the Ecological Society of America 101:e01742.

[eap2348-bib-0044] Mourad, T. M., A. F. McNulty, D. Liwosz, K. Tice, and F. Abbott. 2018. The role of a professional society in broadening participation in science: a national model for increasing persistence. BioScience 68:715–721.

[eap2348-bib-0045] National Center for Science and Engineering Statistics (NCSES) . 2021. Women, minorities and persons with disabilities in science and engineering report. National Science Foundation, Alexandria, VA, USA. https://ncses.nsf.gov/pubs/nsf21321/

[eap2348-bib-0046] NSF . 2020. National Science Foundation Broader Impacts criterion. https://www.nsf.gov/pubs/policydocs/pappg20_1/pappg_2.jsp#IIC2d

[eap2348-bib-0047] O'Connell, K. E. B., K. Hoke, and R. Nilson. 2018. Report from the field on the design, outcomes and assessment of undergraduate field experiences. Technical report. Oregon State University, Corvallis, Oregon, USA.

[eap2348-bib-0048] Ortega, S., A. Flecker, K. Hoffman, L. Jablonski, J. Johnson‐White, M. Jurgensen‐Armstrong, R. Kimmerer, M. Poston, A. Socha, and J. Taylor. 2006. Women and minorities in ecology II (WAMIE II) committee report. https://www.esa.org/esa/wpcontent/uploads/2012/12/wamieReport2006.pdf

[eap2348-bib-0049] Otto, I. M., D. Reckien, C. P. O. Reyer, R. Marcus, V. LeMasson, L. Jones, A. Norton, and O. Serdicany. 2017. Social vulnerability to climate change: a review of concepts and evidence. Regional Environmental Change. 17:1651–1662.

[eap2348-bib-0050] Palumbo, M. J., S. A. Johnson, F. M. Mundim, A. Lau, A. C. Wolf, S. Arunachalam, O. Gonzalez, J. L. Ulrich, A. Washuta, and E. M. Bruna. 2012. Harnessing smartphones for ecological education, research and outreach. Bulletin of the Ecological Society of America 93:390–393.

[eap2348-bib-0051] Pearson, A. R., and J. P. Schuldt. 2018. A diversity science approach to climate change. Psychology and Climate Change:95–124. 10.1016/B978-0-12-813130-5.00005-9

[eap2348-bib-0052] Rainey, K., Dancy, M., R. Mickelson, E. Stearns, and S. Moller. 2018. Race and gender differences in how sense of belonging influences decisions to major in STEM. International Journal of STEM Education 5:10. 30631700 10.1186/s40594-018-0115-6 PMC6310405

[eap2348-bib-0053] Reiners, W. A. 2016. The Cowles‐Cooper tradition under Murray F. Buell: a personal retrospective. Bulletin of the Ecological Society of America 97:291–300.

[eap2348-bib-0054] Reiners, W. A., D. S. Reiners, and J. A. Lockwood. 2013. Traits of a good ecologist: what do ecologists think? Ecosphere 4:1–22.

[eap2348-bib-0055] Russ, A., S. J. Peters, M. E. Krasny, and R. C. Stedman. 2015. Development of ecological place meaning in New York City. Journal of Environmental Education 46:73–93.

[eap2348-bib-0056] Singer, S. 2019. Six decades of the National Science Foundation's commitment to undergraduate research. Scholarship and practice of undergraduate research 3:16–20.

[eap2348-bib-0057] Skrip, M. 2015. Crafting and evaluating broader impact activities: a theory‐based guide for scientists. Frontiers of Ecology and the Environment 13:273–279.

[eap2348-bib-0058] Smith, M. K., C. Walsh, N. G. Holmes, and M. M. Summers. 2019. Using the Ecology and Evolution‐Measuring Achievement and Progression in Science assessment to measure student thinking across the Four‐Dimensional Ecology Education framework. Ecosphere 10:e02873.

[eap2348-bib-0059] Taylor, D. E. 2017. Racial and ethnic differences in the students' readiness, identity, perceptions of institutional diversity and desire to join the environmental workforce. Journal of Environmental Studies and Sciences 8:152–168.

[eap2348-bib-0060] Tewksbury, J. J., et al. 2014. Natural history's place in science and society. BioScience 64:300–310.

[eap2348-bib-0061] Thompson, S. K., C. J. Neill, E. Wiederhoeft, and S. Cotner. 2016. A model for a course‐based undergraduate research experience (CURE) in a field setting. Journal of Microbiology and Biology Education 17:469–471. 28101276 10.1128/jmbe.v17i3.1142 PMC5134953

[eap2348-bib-0062] Torres, L. E., and B. Bingham. 2008. Fixing the leaky pipe: increasing recruitment of underrepresented groups in ecology. Frontiers in Ecology and the Environment 6:554–555.

[eap2348-bib-0063] Walton, G. M., and G. L. Cohen. 2007. A question of belonging: race, social fit, and achievement. Journal of Personality and Social Psychology 92:82–96. 17201544 10.1037/0022-3514.92.1.82

[eap2348-bib-0064] Walton, G. M., and G. L. Cohen. 2011. A brief social‐belonging intervention improves academic and health outcomes of minority students. Science 331:1447–1451. 21415354 10.1126/science.1198364

[eap2348-bib-0065] Yeager, D. S., and C. S. Dweck. 2012. Mindsets that promote resilience: when students believe that personal characteristics can be developed. Educational Psychologist 47:302–314.

