# Supplementary material for: Developing the ecological scientist mindset among underrepresented students in ecology fields
Source: Ecol Appl. 2021 Jun 28;31(6):e02348. doi: 10.1002/eap.2348 (PMC8459229; doi:10.1002/eap.2348)
Supplement: Supplementary file 1 — Appendix S1 [file EAP-31-e02348-s001.pdf]

Supporting Information. Bowser, G. and C.R. Cid. 2021. Developing the ecological scientist mindset among underrepresented students in ecology fields. Ecological Applications.

Appendix S1

Apple, J., J. Lemus, and S. Semken. 2014. Teaching geoscience in the context of culture and place. Journal of Geoscience Education 62:1–4. doi:10.5408/1089-9995-62.1.1

Baker, B. 2000. Recruiting minorities to the biological sciences. BioScience 50:191–195.

Chamany, K, D. Allen, and K. Tanner. 2008. Making biology learning relevant to students: integrating people, history and context into college biology teaching. CBE-Life Sciences Education 7:267–278.

Fisher, A. J., R. Mendoza-Denton, C. Patt, I. Young, A. Eppig, R. L. Garrell, D. C. Rees, T. W. Nelson, and M. A. Richards. 2019. Structure and belonging: pathways to success for underrepresented minority and women PhD students in STEM Fields. PLOS ONE. doi:10.1371/journal.pne.0209279

Foster, J. J., C. Bennett, E. J. Sterling, and N. Bynum. 2011. Fostering the development of conservation leadership at minority-serving institutions. Fisheries 36:461–463.

Gopalan, M., and S. T. Brady. 2019. College students' sense of belonging: a national perspective. Educational Researcher doi:10.3102/0013189X19897622

Hasmann, R. Slotow, J. K. Burns, and E. Di Minin. 2016. The ecosystem service of sense of place: benefits for human well-being and biodiversity conservation. Environmental Conservation 43:117–127.

Haynes, N., S. K. Jacobson, and D. M. Wald. 2015. A life-cycle analysis of minority underrepresentation in natural resource fields. Wildlife Society Bulletin 39:228–238. doi:10.1002/wsb.525

McDaris, J. R., C. A. Manduca, E. R. Iverson, and C. H. Orr. 2017. Looking in the right places: minority-serving institutions as sources of diverse earth science learners. Journal of Geoscience Education 65:407–415.

Masterson, V. A., R. C. Stedman, J. Enqvist, M. Tengö, M. Giusti, D. Wahl, and U. Svedin. 2017. The contribution of sense of place to social-ecological systems research: a review and research agenda. Ecology and Society 22. doi:10.5751/ES-08872-220149

Masterson, J. P. Enqvist, R. C. Stedman, and M. Tengö. 2019. Sense of place in social-ecological systems: from theory to empirics. Sustainability Science 14:555–564.

Morales, N., and S. K. Jacobson. 2019. Assessing natural resource internships: a social cognitive analysis of national diversity programs. Applied Environmental Education & Communication 18:96–112. doi:10.1080/1533015X.2018.1435320

Puniwai-Ganoot, N., S. Ziegler-Chong, R. Ostertag, and M. U. Ching. 2018. Mentoring Pacific Island students for conservation careers. Scholarship and Practice of Undergraduate Research 1:25–32.

Semken, S. 2005. Sense of place and place-based introductory geoscience teaching for American Indian and Alaska native undergraduates. Journal of Geoscience Education 53:149–157.

Semken, S., and C. B. Freeman. 2008. Sense of place in the practice and assessment of place-based science teaching. Science Education 92:1042–1057. doi:10.1002/sce.20279

Semken, E. G. Ward, S. Moosavi, and P. W. U. Chinn. 2017. Place-based education in geoscience: theory, research, practice and assessment. Journal of Geoscience Education 65:542–562.

Sherman-Morris, K., and K. S. McNeal. 2016. Understanding perceptions of the geosciences among minority and nonminority undergraduate students. Journal of Geoscience Education 64:147-156.

Wilson, A. E., J. L. Pollock, I. Billick, C. Domingo, E. G. Fernandez-Figueroa, E. S. Nagi, T. D. Stewry, and A. Summers. 2018. Assessing science training programs: structured undergraduate research programs make a difference. BioScience 68:529–534.

Yaffe, K., C. Bender, and L. Sechrest. 2014. How does undergraduate research experience impact career trajectories and level of career satisfaction: a comparative survey. Journal of College Science Teaching 44:25–33.
